# Correlates of change in accelerometer-assessed total sedentary time and prolonged sedentary bouts among older English adults: results from five-year follow-up in the EPIC-Norfolk cohort

**DOI:** 10.18632/aging.202497

**Published:** 2021-01-11

**Authors:** Dharani Yerrakalva, Samantha Hajna, Katrien Wijndaele, Kate Westgate, Kay-Tee Khaw, Nick Wareham, Simon J Griffin, Soren Brage

**Affiliations:** 1 Department of Public Health and Primary Care, University of Cambridge School of Clinical Medicine, Cambridge, UK; 2 MRC Epidemiology Unit, University of Cambridge, School of Clinical Medicine, Cambridge, UK

**Keywords:** sedentary, correlates, older adults, epidemiology, bouts

## Abstract

Background: Development of effective strategies to reduce sedentary time among older adults necessitates understanding of its determinants but longitudinal studies of this utilising objective measures are scarce.

Methods: Among 1536 older adults (≥60 years) in the EPIC-Norfolk study, sedentary time was assessed for seven days at two time-points using accelerometers. We assessed associations of change in total and prolonged bouts of sedentary time (≥ 30 minutes) with change in demographic and behavioural factors using multi-level regression.

Results: Over follow-up (5.3±1.9 years), greater increases in total sedentary time were associated with older age, being male, higher rate of increase in BMI, lower rate of increase in gardening (0.5 min/day/yr greater sedentary time per hour/week/yr less gardening, 95% CI 0.1, 1.0), a lower rate of increase in walking (0.2 min/day/yr greater sedentary time per hour/week/yr less walking, 95% CI 0.1, 0.3) and a higher rate of increase in television viewing. Correlates of change in prolonged sedentary bouts were similar.

Conclusion: Individuals in specific sub-groups (older, male, higher BMI) and who differentially participate in certain behaviours (less gardening, less walking and more television viewing) but not others increase their sedentary time at a higher rate than others; utilising this information could inform successful intervention content and targeting.

## INTRODUCTION

Excess sedentary time increases adults’ risk of type 2 diabetes, cardiovascular disease, cancer, poor physical function, poor quality of life, and premature mortality. [1–3] Time accrued in prolonged sedentary bouts is thought to be particularly harmful. [4–6] Describing changes in sedentary time and their correlates will clarify whether older adults have the capacity to change and, if so, to what extent. [7] It will also enable deeper understanding of patterns and correlates of sedentary time, which facilitates greater specificity in intervention development. Despite sedentary time occurring in complex behavioural settings, research on correlates has not reflected this complexity [8].

Importantly, no prospective studies have quantified associations of baseline values and changes in behavioural correlates with changes in sedentary time. As interventions increasingly target both reducing sitting activities and replacing this with physical activities, it is important to have an evidence-base for the choice of activity to target. [9] If changes in specific behaviours (e.g. walking, housework, TV viewing) are associated with increases in sedentary time, this may indicate which context-specific behaviours could be targeted in future interventions. As interventions to reduce sedentary time have so far not been successful in maintaining behaviour change beyond a year, observational evidence of this type is key to designing more effective strategies. [10, 11].

Studies of sociodemographic correlates have also been neglected, despite the fact they may help us specify who we might most usefully target. This type of studies has been limited by mostly cross-sectional study designs and the use of self-reported measures of sedentary time [12–17], with only a handful of exceptions [18–20]. Cross-sectional socio-demographic correlates of increased sedentary time include older age, male sex, retirement, lower educational attainment, and poorer self-rated health. [21].

It is particularly important to examine these associations in the neglected group of older adults. Previously observed associations in other groups may not apply given that older adults exhibit different physiology and anthropometry (for example decreases in bone and muscle mass, and increases in adiposity with age) [22] to their younger counterparts, affecting which activities they are able and chose to do. Further, older adults are more likely to be close to retirement [23], which may influence behaviours. For retired individuals, the choice of activities is not limited by occupation (eg having to sit at a desk), and so they may have more opportunity and therefore capacity for change. Given older adults are also at higher risk of conditions (e.g. diabetes, coronary heart disease) where routine enquiry and advice about activity levels are currently being delivered in primary care settings [24], it is important that we expand the evidence that underpins interventions tailored to older adults.

We aimed to estimate, in a population-based sample of older English adults, five-year changes in objectively-assessed sedentary time and prolonged sedentary bouts. Further, we sought to be the first study to identify whether these changes were associated with baseline socio-demographic and behavioural factors and changes in these factors over time.

## RESULTS

Of the 1,587 participants who had accelerometer data at both baseline and follow-up health checks, 51 were excluded due to having <4 valid days of data (42 excluded) or having >19 hr/day average wear-time (9 excluded), leaving a total of 1,536 (96.8%) participants. Sensitivity analyses utilising complete case analysis included 953 and 856 participants for baseline and change in correlate analyses, respectively. All results referred to in the text relate to maximally adjusted models unless otherwise stated.

### Descriptive characteristics

Participants were on average 68.6 years old (SD=6.2) (Table 1). At baseline, 23.6% were employed. Participants accumulated an average of 9.2 hr/day of sedentary time (SD=1.5) at baseline and 9.6 hr/day (SD=1.4) at follow-up and 3.2 hr/day in prolonged sedentary bouts (SD=1.7) at baseline and 3.9 hr/day (SD=1.8) at follow-up. Average daily wear time was 14.5 hours (SD=1.0) at baseline and 14.3 hours (SD=1.0) at follow-up. Mean daily sedentary time increased 5.7 min/day/yr (SD=17.1) and prolonged sedentary bout time increased 8.3 min/day/yr (SD 20.6). Average follow-up time was 5.3 years (SD=1.9). Included and excluded participants were socio-demographically similar (data not shown).

**Table 1:** Baseline Characteristics n= 1,536.

**Characteristics**		***Frequency***	**Percent (%)**
**Sex**	Female	685	44.6
	Male	851	55.4
**Age (years)**	60-<65	528	34.4
	65-<70	422	27.5
	70-<75	331	21.6
	75-<80	191	12.4
	80-<85	48	3.1
	≥85	16	1.0
**Ethnicity**	White	1,526	99.3
	Black Other	1	0.1
	Indian	1	0.1
	Missing	8	0.5
**Occupational Classification**	Professional	136	8.9
	Manager	669	43.6
	Skilled non-manual	207	13.5
	Skilled manual	317	20.5
	Semi-skilled	165	10.7
	Non-skilled	30	1.9
	Missing	14	0.9
**Employed**	No	1164	75.7
	Yes	359	23.3
	Missing	16	1.0
**Further Education level**	O-level or lower	536	34.9
	A-level or higher	1000	65.1
**Smoking Status**	Current	39	2.5
	Former	691	45.5
	Never	806	52.0
**History of Diabetes**	No	1489	97.5
	Yes	38	2.5
**History of Myocardial Infarction**	No	1490	97.0
	Yes	46	3.0
**Body Mass Index (kg/m^2^)**	<18.5	8	0.5
	18.5-<25	565	36.8
	25-<30	695	45.3
	30-<35	213	13.9
	≥35	55	3.5

### Correlates of changes in sedentary time

### Demographic correlates

### Total sedentary time

Greater rate of increase in total sedentary time was associated with older age (Table 2A, 0.6 min/day/yr per year of age, 95% CI 0.4, 0.7), being male (1.6 min/day/yr, 95% CI 0.1,3.0), higher BMI (0.3 min/day/yr per kg/m^2^, 95% CI 0.2, 0.5) and higher rate of increase in BMI (Table 3; every 1 kg/m^2^ per year increase in BMI was associated with 6.3 min/day/yr more sedentary time, 95% CI 4.4,8.2). Greater rate of increase in total sedentary time was associated with urban versus rural living (Table 2A; 1.9 min/day/yr, 95% CI 0.2, 3.7) and non-skilled versus professional occupational class (6.0 min/day/yr, 95% CI 0.01, 11.4). There was a trend towards greater rate of increase in sedentary time for those who remained retired versus remained employed (Table 3, 2.2 min/day/yr, 95% CI -0.2, 4.6). There was no difference between those who remained employed and those who became retired.

**Table 2A:** Association of baseline demographic correlates with changes in total sedentary time and prolonged sedentary bouts (n=1,536).

**Baseline characteristic**	**Category/Unit**	**Total sedentary time (Min/day/yr)**						**Prolonged sedentary bouts (Min/day/yr)**					
		**Model 1^a^**		**Model 2^b^**		**Model 3^c^**		**Model 1^a^**		**Model 2^b^**		**Model 3^c^**	
		**β**	**95% CI**	**β**	**95% CI**	**β**	**95% CI**	**β**	**95% CI**	**β**	**95% CI**	**β**	**95% CI**
**Sex**	Male (ref)												
	Female	**-1.9**	**-3.3, -0.4**	**-1.5**	**-2.9, -0.08**	**-1.6**	**-3.0, -0.1**	**-3.2**	**-5.1, -1.3**	**-2.8**	**-4.7, -0.07**	**-2.5**	**-4.4, -0.6**
**Age**	Per year of age	**0.6**	**0.4, 0.7**	**0.6**	**0.4, 0.7**	**0.6**	**0.4, 0.7**	**0.8**	**0.6, 0.9**	**0.8**	**0.6, 0.9**	**0.6**	**0.2, 0.8**
**Employment**	Yes (ref)												
	No	**3.0**	**1.3, 4.6**	0.8	-1.0, 2.6	0.7	-1.0, 2.5	**2.3**	**0.1, 4.5**	-0.6	-2.9, 1.7	-0.6	-2.9, 1.7
**Education level**	O-level or less (ref)												
	A-level or above	-0.7	-2.1, 0.8	-0.4	-1.8, 1.0	-2.7	-1.7, 1.2	0.7	-1.2, 2.6	0.8	-1.0, 2.7	0.8	-1.1, 2.7
**Smoking status**	Current (ref)												
	Former	1.7	-2.7, 6.2	-0.5	-4.9, 3.9	-0.8	-5.2, 3.6	3.7	-2.1, 9.6	0.9	-4.8, 6.6	0.3	-5.4, 6.1
	Never	1.2	-3.3, 5.6	-0.8	-5.1, 3.6	-0.7	-5.0, 3.6	2.4	-3.4, 8.3	-0.06	-5.7, 5.6	-0.3	-6.0, 5.4
**Body Mass Index**	Per kg/m^2^	**0.3**	**0.1, 0.5**	**0.3**	**0.2, 0.5**	**0.3**	**0.2, 0.5**	**0.3**	**0.1, 0.5**	**0.4**	**0.2, 0.6**	**0.4**	**0.2, 0.6**
**Occupational classification**	Professional (ref)												
	Manager	1.9	-0.7, 4.4	1.8	-0.7, 4.3	1.6	-0.9, 4.1	0.4	-3.0, 3.7	0.3	-3.0, 3.5	0.1	-3.1, 3.4
	Skilled non-manual	0.9	-2.1, 3.9	1.0	-2.0, 3.9	0.5	-2.5, 3.4	0.2	-3.7, 4.2	0.4	-3.4, 4.3	0.4	-3.4, 4.3
	Skilled manual	1.2	-1.6, 4.0	1.0	-1.7, 3.8	0.8	-2.0, 3.5	0.04	-3.6, 3.7	-0.4	-3.9, 3.2	-0.4	-4.0, 3.2
	Semi-skilled	1.8	-1.4, 4.9	1.6	-1.5, 4.6	1.1	-2.0, 4.35	-0.4	-4.6, 3.7	-0.6	-4.6, 3.4	-0.6	-4.6, 3.6
	Non-skilled	**7.0**	**1.5, 12.4**	**6.6**	**1.3, 11.9**	**6.0**	**0.007, 11.4**	2.6	-4.6, 9.8	1.9	-5.1, 8.8	2.0	-5.0, 9.1
**Urban-rural status**	City, town or fringe (ref)												
	Village, hamlet or isolated dwelling	**-1.9**	**-3.6, -0.1**	**-1.9**	**-3.6, -0.2**	**-1.9**	**-3.7, -0.2**	-1.0	-3.2, 1.3	-0.9	-3.1, 1.3	-1.0	-3.2, 1.2

^a^Model 1 was adjusted for season and wear time at baseline and follow-up, and baseline total sedentary time.

^b^Model 2 was the same as model 1 plus mutually adjusted for age and sex.

^c^Model 3 was the same as Model 2 plus mutually adjusted for potential socioeconomic and environmental confounders (occupational class, educational level, job status, urban-rural status, smoking status, BMI).

**Table 3:** Association of change in correlates with change in total sedentary time and prolonged sedentary bouts (n=1536).

**Change in correlate**	**Category/unit**	**Total sedentary time (Min/day/yr)**						**Prolonged sedentary bouts (Min/day/yr)**					
		**Model 1**		**Model 2**		**Model 3**		**Model 1**		**Model 2**		**Model 3**	
		**β**	**95% CI**	**β**	**95% CI**	**β**	**95% CI**	**β**	**95% CI**	**β**	**95% CI**	**β**	**95% CI**
**Employment status**	Remains employed (ref)												
	Becomes employed	1.3	-5.3, 7.8	0.8	-5.6, 7.2	0.9	-5.5, 7.3	1.6	-7.0, 10.3	1.0	-7.4, 9.3	0.9	-7.5, 9.3
	Remains retired	**4.4**	**2.0, 6.7**	**2.5**	**0.08, 4.8**	2.2	-0.2, 4.6	**4.0**	**0.9, 7.1**	0.4	-1.7, 4.5	1.3	-1.9, 4.4
	Becomes retired	2.2	-0.7, 5.1	2.5	-0.3, 5.3	2.3	-0.6, 5.1	2.9	-0.9, 6.7	3.3	-0.4, 7.0	3.1	-0.6, 6.8
**Body Mass Index**	Per kg/m^2^/yr	**5.0**	**3.1, 6.9**	**5.6**	**3.7, 7.5**	**6.3**	**4.4, 8.2**	**4.6**	**2.0, 7.1**	**5.6**	**3.1, 8.0**	**5.5**	**3.1, 8.0**
**Walking**	Per hour/week/yr	**-0.5**	**-0.8, -0.2**	**-0.5**	**-0.8, -0.3**	**-0.2**	**-0.3, -0.06**	-0.2	-0.6, 0.2	-0.3	-0.6, 0.1	-0.1	-0.3, 0.02
**Cycling**	Per hour/week/yr	1.2	-0.02, 2.4	**1.2**	**0.05, 2.3**	0.7	-0.7, 2.0	**1.8**	**0.3, 3.4**	**1.9**	**0.4, 3.4**	1.2	-0.6, 2.9
**Gardening**	Per hour/week/yr	-0.4	-0.8, 0.06	-0.3	-0.7, 0.2	**-0.5**	**-1.0, -0.1**	**-0.6**	**-1.1, -0.002**	-0.4	-1.0, 0.10	**-0.8**	**-1.4, -0.2**
**Housework**	Per hour/week/yr	-0.1	-0.3, 0.1	-0.1	-0.3, 0.1	-0.1	-0.3, 0.2	-0.1	-0.4, 0.2	-0.1	-0.4, 0.2	-0.1	-0.5, 0.2
**TV**	Per hour/week/yr	**3.2**	**0.03, 3.3**	**3.4**	**0.4, 6.5**	**4.4**	**1.2, 7.6**	**4.3**	**0.4, 8.2**	**4.3**	**0.4, 8.2**	**5.2**	**1.1, 9.3**

^a^Model 1 was adjusted for season and wear time at baseline and follow-up, and baseline total sedentary time.

^b^Model 2 was the same as model 1 plus mutually adjusted for age and sex.

^c^Model 3 was the same as Model 2 plus mutually adjusted for potential socioeconomic and environmental confounders (occupational class, educational level, job status, urban-rural status, smoking status, BMI).

### Prolonged sedentary bouts

Greater rate of increase in prolonged sedentary bout time was associated with older age (Table 2A, 0.6 min/day/yr per year of age, 95% CI 0.2,0.8), being male (2.5 min/day/yr, 95% CI 0.6,4.4), higher BMI (0.4 min/day/yr per kg/m^2^, 95% CI 0.2,0.6) and with higher rate of increase in BMI (Table 3, every 1kg/m^2^ per year increase in BMI was associated with 5.5 min/day/yr more in prolonged sedentary bouts, 95% CI 3.1,8.0).

### Behavioural correlates

### Total sedentary time

Greater rate of increase in total sedentary time was associated with lower levels of baseline cycling (Table 2B, 0.4 min/day/yr more sedentary time for every hour/week less cycling, 95% CI 0.09, 0.6). This was also the case for winter and summer cycling, separately (Supplementary Table 1A). Greater rate of increase in total sedentary time was associated with less baseline gardening (Table 2B, 0.1 min/day/yr more sedentary time for every hour/week less gardening, 95% CI 0.004 0.2). This was also the case for winter but not summer gardening (Supplementary Table 1A).

**Table 2B:** Association of baseline behavioural correlates with changes in total sedentary time and prolonged sedentary bouts (n=1,536).

**Correlate**	**Category/unit**	**Total sedentary time (Min/day/yr)**						**Prolonged sedentary bouts (Min/day/yr)**					
		**Model 1**		**Model 2**		**Model 3**		**Model 1**		**Model 2**		**Model 3**	
		**β**	**95% CI**	**β**	**95% CI**	**β**	**95% CI**	**β**	**95% CI**	**β**	**95% CI**	**β**	**95% CI**
**Walking**	Per hour/week	0.0	-0.1, 0.04	0.0	-0.1, 0.04	-0.1	-0.2, 0.03	-0.1	-0.2, 0.04	-0.1	-0.2, 0.03	-0.1	-0.2, 0.05
**Cycling**	Per hour/week	**-0.3**	**-0.6, -0.07**	**-0.4**	**-0.6, -0.1**	**-0.4**	**-0.6, -0.09**	**-0.6**	**-0.9, -0.2**	**-0.6**	**-0.9, -0.2**	**-0.6**	**-0.9, -0.2**
**Gardening**	Per hour/week	0.0	-0.1, 0.1	**-0.1**	**-0.2, -0.01**	**-0.1**	**-0.2, -0.004**	0.0	-0.2, 0.1	**-0.2**	**-0.3, -0.02**	**-0.2**	**-0.3, -0.009**
**Housework**	Per hour/week	0.0	-0.1, 0.05	0.0	-0.05, 0.08	0.0	-0.05, 0.08	0.0	-0.1, 0.03	0.0	-0.09, 0.08	0.0	-0.08, 0.09
**Dog walking**	No (ref)												
	Yes	-0.6	-2.7, 1.5	-0.5	-2.6, 1.5	-0.6	-2.7, 1.4	-0.2	-2.7, 2.3	0.2	-2.2, 2.6	0.2	-2.2, 2.6
**Transport method <1 mile**	Car (ref)												
	Walk	1.7	-0.5, 3.9	1.0	-1.2, 3.1	1.1	-1.01, 3.3	2.9	-0.1, 5.7	1.9	-0.8, 4.7	2.3	-0.5, 5.0
	Public transport	2.0	-6.2, 10.3	0.9	-7.1, 9.0	0.7	-7.3, 8.7	1.5	-9.4, 12.4	0.4	-10.3, 11.0	0.3	-10.3, 11.0
	Cycle	1.4	-2.3, 5.2	0.5	-3.2, 4.1	0.8	-2.9, 4.5	0.3	-4.8, 5.3	-0.8	-5.8, 4.2	-0.1	-5.2, 4.9
**Transport method 1-5 miles**	Car (ref)												
	Walk	0.9	-1.6, 3.4	0.6	-1.8, 3.0	0.7	-1.8, 3.1	0.6	-2.6, 3.8	0.6	-1.9, 3.1	0.8	-2.4, 4.0
	Public transport	1.2	-1.3, 3.7	0.5	-2.0, 3.0	0.2	-2.4, 2.7	0.9	-2.3, 4.1	0.5	-2.0, 2.9	-0.3	-3.5, 2.9
	Cycle	0.9	-1.8, 3.6	0.3	-2.3, 2.9	0.3	-2.3, 2.9	-2.8	-6.1, 0.5	0.3	-2.2, 2.8	-3.0	-6.3, 0.2
**Transport method >5 miles**	Car (ref)												
	Walk	-1.5	-12.7, 9.8	-3.2	-14.5, 8.2	-3.4	-14.6, 7.8	-5.9	-20.1, 8.4	-7.5	-21.6, 6.6	-7.0	-20.8, 6.8
	Public transport	0.6	-2.6, 3.8	-0.6	-3.7, 2.5	-0.8	-4.0, 2.3	-2.6	-6.7, 1.5	-4.3	-8.2, 6.6	**-4.3**	**-8.3, -0.3**
	Cycle	-1.7	-9.6, 6.3	-2.7	-10.4, 5.3	-3.4	-11.1, 4.4	-2.6	-12.8, 7.5	-3.7	-13.7, 6.2	-4.5	-14.4, 5.4
**TV**	Per hour/week	0.4	-0.1, 1.0	0.4	-0.2, 0.9	0.2	-0.4, 0.7	0.3	-0.4, 1.0	0.2	-0.4, 0.9	0.2	-0.5, 0.9
**Radio**	≤Several times/yr (ref)												
	Several times/month	-0.4	-2.9, 2.2	-0.5	-2.9, 2.0	-0.7	-3.2, 1.7	0.0	-3.3, 3.3	-0.2	-3.4, 3.0	-0.4	-3.6, 2.8
	≥Several times/week	-1.1	-3.0, 0.7	-1.5	-3.3, 0.3	-1.4	-3.2, 0.4	-0.2	-2.6, 2.3	-0.7	-3.1, 1.7	-0.7	-3.1, 1.7
**Newspaper**	≤Several times/yr (ref)												
	Several times/month	2.5	-1.0, 6.0	2.6	-0.8, 5.9	2.8	-0.6, 6.1	-0.3	-4.7, 4.2	0.1	-4.2, 4.4	0.1	-4.3, 4.4
	≥Several times/week	2.9	-0.1, 5.7	1.9	-0.8, 4.7	2.1	-0.6, 4.8	2.2	-1.5, 5.8	1.0	-2.5, 4.5	1.0	-2.5, 4.5
**Reading books**	≤Several times/yr (ref)												
	Several times/month	-1.2	-3.7, 1.4	-0.8	-3.27, 1.70	-0.6	-3.1, 1.9	-0.6	-4.0, 2.8	0.2	-3.1, 3.5	0.2	-3.1 3.5
	≥Several times/week	-0.8	-2.5, 0.8	-0.6	-2.3, 1.0	-0.4	-2.1, 1.3	-1.0	-3.2, 1.2	-0.5	-2.7, 1.6	-0.6	-2.8, 1.6
**Computer use**	Per hour/week	**-1.5**	**-0.3, -2.7**	-0.4	-1.7, 0.8	-0.3	-1.5, 1.0	-1.3	-2.9, 0.2	-0.1	-1.7, 1.5	-0.4	-2.0, 1.3

^a^Model 1 was adjusted for season and wear time at baseline and follow-up, and baseline total sedentary time.

^b^Model 2 was the same as model 1 plus mutually adjusted for age and sex.

^c^Model 3 was the same as Model 2 plus mutually adjusted for potential socioeconomic and environmental confounders (occupational class, educational level, job status, urban-rural status, smoking status, BMI).

Greater rate of increase in total sedentary time was also associated with lower rate of increase in gardening time (Table 3, every hour/week decrease in gardening per year was associated with 0.5 min/day/yr more total sedentary time, 95% CI 0.1, 1.0) as well as summer gardening time but not winter (Supplementary Table 1B). Greater rate of increase in total sedentary time was associated with lower rate of increase in walking time (Table 3, every hour/week decrease in walking per year was associated with 0.2 min/day/yr more total sedentary time, 95% CI 0.06, 0.3). Greater rate of increase in total sedentary time was associated with higher rate of increase in TV viewing time (Table 3, every hour/week increase in TV viewing per year was associated with 4.4 min/day/yr more total sedentary time, 95% CI 1.2, 7.6).

### Prolonged sedentary bouts

Greater rate of increase in prolonged bout time was associated with less baseline cycling (Table 2B, 0.6 min/day/yr greater prolonged bout time per hour/week less cycling, 95% CI 0.2, 0.9), as well as less summer and winter cycling (Supplementary Table 1B). Greater rate of increase in prolonged bout time was associated with less gardening at baseline (Table 2B, 0.2 min/day/yr greater prolonged bout time per hour/week less gardening, 95% CI 0.009, 0.3), and less winter but not summer gardening (Supplementary Table 1B). Greater rate of increase in prolonged bout time was also associated with lower rate of increase in gardening (Table 3, every hour/week decrease in gardening per year was associated with 0.8 min/day/yr more prolonged bout time, 95% CI 0.2, 1.4), and summer but not winter gardening (Supplementary Table 1B). There was no association with walking, housework, dog walking, change in walking, or change in housework (Table 2B).

Greater rate of increase in prolonged bout time was associated with higher rate of increase in TV viewing time (Table 3, every hour/week increase in TV viewing per year was associated with 5.2 min/day/yr more prolonged bout time, 95% CI 1.1, 9.3). There were no associations between transport-related correlates and change in total sedentary time. Greater rates of increase in time in prolonged sedentary bouts trended towards an association with car use compared to cycling for journeys 1-5 miles (3.0 min/day/yr, 95% CI -0.2, 6.3) and public transport for journeys >5 miles (4.3 min/day/yr, 95% CI 0.3, 8.3).

### Sensitivity analyses

There were no important differences in results when using complete-case analyses versus multiple imputation analyses (Supplementary Tables 2A–2C). The results were also comparable when using a valid day threshold of ≥5 days versus ≥4 days (data not shown).

## DISCUSSION

In this cohort of English older adults, individuals accumulated large amounts of total sedentary time and prolonged bout time which increased over time. This is the first study to examine the association of changes in total sedentary time and prolonged sedentary bout time with a wide range of demographic and behavioural correlates. We found that a greater rate of increase in total sedentary time and/or prolonged bout time was associated with older age, being male, higher BMI, higher rate of increase in BMI, urban dwelling, and being classified as non-skilled. We also found that a greater rate of increase in total sedentary time was associated with less winter and summer cycling, less winter gardening, lower rates of increase in walking time and gardening time, and a higher rate of increase in TV viewing time.

This is important because previous sedentary time interventions have targeted a multitude of activities [7, 11, 25, 26] both to reduce sedentary time and replace it with physical activity, with no clear conclusion on which context-specific behaviours it is most effective to target. [27] Our findings suggest that targeting particular activities (improvements in walking and summer gardening, and reductions in TV viewing time) might be more successful than others in preventing increases in total sedentary time and prolonged bout time. The advantage of understanding which context-specific behaviour to target (e.g. TV time), is that specific environment cues can be used to encourage behaviour change and habit formation (e.g. cue card to take standing break by TV remote) [28].

The fact that greater rate of increase in total sedentary time and prolonged sedentary bouts was associated with baseline BMI and a higher rate of increase in BMI is important for future intervention planning. It is plausible that those with larger BMIs may be more sedentary than their lower BMI counterparts given that activities require a greater metabolic effort for these individuals. As we cannot rule out reverse causality, it is possible that an intervention to prevent increase in sedentary time may concurrently limit increases in BMI and vice versa, thus BMI and sedentary time might be jointly targeted.

Non-skilled and urban-dwellers could also be specifically targeted. Future work should examine why rural-dwellers are less sedentary and these data could inform intervention content for urban-dwellers (e.g. walking-friendly urban design, parks in cities) [29]. Further work is also needed to investigate why individuals in non-skilled occupational class demonstrate high rates of increases in sedentary time.

### Findings in the context of the literature

This is the first study to examine whether change in accelerometer-assessed sedentary time is associated with baseline behavioural factors and changes in these factors over time. Very few studies have examined these associations with socio-demographic factors. In a US cohort (n=962) of middle-aged adults (45.0±3.5 year at baseline), Gabriel et al. [20] found that accelerometer-assessed sedentary time increased by 37.9 minutes/day (SE 3.7) over 10 years. We found, as expected in an older cohort, a higher rate of increase in sedentary time (5.7 min /day/yr). We extend previous work in the EPIC-Norfolk cohort, in which older age, higher BMI, and urban dwelling in women were found to be correlates of increases in sedentary time. [19] To our knowledge, only one other longitudinal study has examined change in accelerometer-assessed bouted sedentary time (≥30 minutes). [18] Yonemoto et al. [18] examined change in total and bouted sedentary time over three years in 1,151 Japanese adults aged ≥40 years. They found that accelerometer-assessed total sedentary time increased by a median of 14.8 minutes in men and 13.5 minutes in women, and that bouted sedentary time (≥30 mins) increased by a median of 15.3 minutes in men and 10.5 minutes in women but did not undertake correlate analysis. These estimates are comparable to the increases we report here.

Strengths of our study include utilisation of data from EPIC-Norfolk, a large population-based cohort of older adults in which objective measures of sedentary time were available at two time points. These data allowed us to overcome the two main criticisms of research in this area, namely subjective measure use and cross-sectional design. Some limitations should also be noted. Participants who wore accelerometers may have changed their behaviour as a result of being measured, though changes are unlikely to be sustained over five days. [30, 31] Secondly, hip-mounted accelerometers are unable to discriminate standing still and sitting, aren’t sensitive to upper body movement and water-based activities may also be classified as non-wear time given that participants were asked to remove their monitors during such activities. We used a non-wear algorithm supported by the existing literature [32, 33] with non-wear time threshold defined as ≥90 minutes. Thirdly, it is possible that uniaxial hip-mounted accelerometers underestimate sedentary activity in comparison to thigh-mounted tri-axial accelerometers which more accurately distinguish between sitting/reclining and upright postures. [34–36] Like other cohort studies, this study was subject to potential healthy volunteer bias and attrition and therefore included participants are likely to be healthier than the wider EPIC-Norfolk cohort and the general population [37]. Finally, some of the variables in our dataset had missing data up to 25%, leaving the possibility of missing data bias. Our use of multiple imputation with this level of missing data is appropriate according to the literature [38–40]. We used multiple imputation analyses, with the assumption that data were missing at random, and a sensitivity analyses utilising complete case analysis to deal with this [38–40].

## CONCLUSIONS

We found that older English adults spent significant amounts of their waking hours sedentary, and this sedentary time increased as participants aged. While existing interventions have aimed to reduce total sedentary time, we found that time in prolonged sedentary bouts also increased. Therefore, preventing increases in bouted sedentary time may also be important. We found that specific sub-groups (older, male, higher BMI, larger increases in BMI, urban-dwellers, non-skilled occupational class) and particular behaviours (decreases in walking time and gardening, and increases in TV viewing time) were associated with greater increases in sedentary time. This information should inform the development and targeting of future interventions aimed at reducing total sedentary time and time spent in prolonged sedentary bouts in older adults.

## MATERIALS AND METHODS

### Study sample

We utilised data from the EPIC-Norfolk study, a large prospective cohort of adults living in Norfolk (UK) recruited between 1993-1997 from 35 general practices. [41] Participants were similar to the national population sample in Health Survey of England in terms of anthropometry, serum lipids and blood pressure. [37] Four health checks were conducted between 1993-2016. We utilised data from the third (2004-2011) and fourth (2012-2016) health checks (hereafter referred to as baseline and follow-up assessments, respectively) which incorporated objective measures of sedentary time. As UK rates of retirement are high from aged 60 onwards [23] and the United Nations definition of an older person is an adult aged ≥60 [42], we restricted this analysis to adults aged ≥60 at baseline.

### Sedentary time

A total of 8,623 and 5,696 participants ≥60 years old attended the baseline and follow-up assessments, respectively. A sub-sample were invited to wear accelerometers at baseline (n=3,784) and follow-up (n=4,788). We utilised data from individuals who wore accelerometers at both health-checks. Participants were asked to wear accelerometers (Actigraph, Pensacola, USA) on the right hip for 7 days during waking hours and remove them while showering, bathing or swimming. Participants wore a uniaxial accelerometer (GT1M) at baseline and a triaxial accelerometer (GT3X+) at follow-up. Uniaxial monitors were initialised to record activity and step frequency in five-second epochs and triaxial accelerometers were initialised to collect raw acceleration in 100 Hz; both data sources were integrated into 60-second epochs in this analysis. [43] These two models of accelerometers are considered comparable in their measurement of sedentary time. [44, 45] We harmonised the data from the two accelerometers using a method that has been described elsewhere. [19].

Non-wear time was defined as continuous zero counts of ≥90 minutes [46]. Total sedentary time and time in prolonged sedentary bouts (≥30 minutes) were expressed as minutes/day. The threshold to define sedentary time was <100 counts per minute (cpm) [47]. Participants with >19 hours/day of average wear time (indicative of overnight wear) or with <4 days of valid wear time (where criteria for each valid day was >10hours), were excluded. To account for variability in follow-up time, we expressed changes in total sedentary time and change in prolonged sedentary bout time as annual rates of change (min/day/yr of follow-up).

### Correlates

### Demographic correlates

Age, sex, smoking status, body mass index (BMI), occupational classification (*Registrar*-*General's Social Classification),* job status, highest educational level, and urban/rural status were assessed via a self-completed health questionnaire (Table 1). BMI (kg/m^2^) was calculated based on weight and height measurements taken by trained research staff following standard operating procedures. Change in employment status was derived based on change in self-reported job status from baseline to follow-up. Rate of change in BMI was calculated as the difference between baseline and follow-up values divided by time in years between the baseline and follow-up visits (kg/m^2^/yr).

### Behavioural correlates

The behavioural correlates of interest included housework, gardening, cycling, walking, dog walking, TV viewing, computer use, newspaper reading, book reading, and radio listening, and were assessed using a self-completed questionnaire [48, 49]. We calculated average walking, cycling and gardening variables by summing the respective summer and winter variables and dividing by two. Rate of change in walking time, cycling time, gardening time (average across the year, summer, winter), housework time, and TV time were calculated as the difference between values at baseline and follow-up divided by the number of years between health checks (i.e. hours/week/yr). Average daily computer use was calculated by the sum of the average daytime and evening computer use variables, weighted for weekend and weekday responses [(5*sum of day and evening weekday time + 2*sum of day and evening weekend time)/7).

### Statistical analysis

Descriptive statistics were calculated for change in sedentary time and time in prolonged sedentary bouts. Multiple imputation analyses by chained equations (MICE; *mi impute chained function* in STATA) were used to account for missing data. [50] Outcome variables and all covariates identified a priori were included in the imputation models, as well as some auxiliary variables to improve the prediction of missing variables. Given that the models for the two different analyses (baseline correlate analysis and change in correlate analysis) had different variables, we ran separate imputations. As the number of imputed datasets should be at least as great as the percentage of individuals with any missing values [50], we generated 38 and 45 imputed datasets in the baseline and change correlate analyses, respectively.

We fitted our analysis models as follows. We assessed associations between baseline correlates and change in sedentary variables using linear regression models. For walking, cycling and gardening, we firstly included models assessing associations with average time variables (across the year), and then summer and winter time variables separately. For computer use, we assessed associations with total use and then evening and daytime use. We then assessed the association between change in correlates and change in sedentary variables.

We examined associations across three models. Model 1 was adjusted for wear-time and season (since season can affect activity levels [51]) at baseline and follow-up, and baseline total sedentary time. Model 2 was the same as Model 1 plus mutually adjusted for age and sex. Model 3 was the same as Model 2 with mutually adjustment for potential sociodemographic confounders (occupational class, educational level, job status, urban-rural status, smoking status, BMI). For the change in correlates analysis, adjustment for respective baseline correlate was added across all models. All analyses were conducted using STATA 15.0 (StataCorp, TX, USA).

### Sensitivity analyses

Complete-case and multiple imputation analyses were compared to examine how the missing data may have affected the results. We also used the alternative threshold of ≥5 days of valid wear-time to examine how our inclusion criteria (≥4 days) affected the results.

## Supplementary Material

Supplementary Tables
