# Supplementary material for: Correlates of change in accelerometer-assessed total sedentary time and prolonged sedentary bouts among older English adults: results from five-year follow-up in the EPIC-Norfolk cohort
Source: Aging (Albany NY). 2021 Jan 11;13(1):134–49. doi: 10.18632/aging.202497 (PMC7835006; doi:10.18632/aging.202497)
Supplement: Supplementary Tables [file aging-13-202497-s001.pdf]

SUPPLEMENTARY TABLES

Supplementary Table 1A. Association of baseline seasonal and diurnal behavioural correlates with changes in total sedentary time and prolonged sedentary bouts (n=1536).

Correlate (Per hr/week)	Season/diurnal period	Total sedentary time (Min/day/yr)						Prolonged sedentary bouts (Min/day/yr)					
		Model 1		Model 2		Model 3		Model 1		Model 2		Model 3	
		β	95% CI	β	95% CI	β	95% CI	β	95% CI	β	95% CI	β	95% CI
**Walking**	Summer	0.0	-0.1, 0.04	0.0	-0.1, 0.04	0.0	-0.1, 0.04	-0.1	-0.2, 0.04	-0.1	-0.2, 0.03	-0.1	-0.2, 0.04
	Winter	0.0	-0.1, 0.04	-0.1	-0.1, 0.04	0.0	-0.1, 0.04	-0.1	-0.2, 0.04	-0.1	-0.2, 0.04	-0.1	-0.2, 0.05
**Cycling**	Summer	**-0.2**	**-0.4, -0.04**	**-0.2**	**-0.4, -0.04**	**-0.2**	**-0.4, -0.04**	**-0.3**	**-0.6, -0.09**	**-0.3**	**-0.6, -0.09**	**-0.3**	**-0.6, -0.09**
	Winter	**-0.4**	**-0.8, -0.003**	**-0.4**	**-0.8, -0.08**	**-0.4**	**-0.8, -0.04**	**-0.7**	**-1.2, -0.3**	**-0.8**	**-0.3, -0.3**	**-0.8**	**-1.2, -0.3**
**Gardening**	Summer	0.0	-0.06, 0.1	-0.1	-0.1, 0.03	-0.1	-0.1, 0.03	0.0	-0.1, 0.09	-0.1	-0.2, 0.003	-0.1	-0.2, 0.01
	Winter	-0.1	-0.3, 0.03	**-0.3**	**-0.4, -0.10**	**-0.3**	**-0.4, -0.1**	-0.1	-0.2, 0.1	**-0.2**	**-0.4, -0.03**	**-0.2**	**-0.4, -0.03**
**Computer use**	Daytime	**-1.3**	**-2.2, -0.4**	-0.4	-1.3, 0.5	-0.2	-1.2, 0.8	-1.1	-2.3, 0.7	0.0	-1.2, 1.1	-0.3	-1.5, 1.0
	Evening	-0.7	-1.8, 0.5	-0.2	-1.4, 0.9	-0.2	-1.3, 1.0	-0.7	-2.2, 0.8	-0.2	-1.7, 1.3	-0.3	-1.8, 1.3

Supplementary Table 1B. Association of change in seasonal and diurnal behavioural correlates with changes in total sedentary time and prolonged sedentary bouts (n=1536).

Change in correlate (Per hr/week/yr)	Season/diurnal period	Total sedentary time (Min/day/yr)						Prolonged sedentary bouts (Min/day/yr)					
		Model 1		Model 2		Model 3		Model 1		Model 2		Model 3	
		β	95% CI	β	95% CI	β	95% CI	β	95% CI	β	95% CI	β	95% CI
**Walking**	Summer	**-0.4**	**-0.7, -0.2**	**-0.5**	**-0.7, -0.2**	**-0.67,**	**-1.0, -0.4**	-0.2	-0.5, 0.13	-0.2	-0.6, 0.09	-0.5	-0.8, 0.08
	Winter	**-0.5**	**-0.8, -0.2**	**-0.6**	**-0.8, -0.3**	**-0.8**	**-0.08, -0.5**	-0.2	-0.6, 0.2	-0.3	-0.6, 0.1	-0.5	-0.9, 0.06
**Cycling**	Summer	0.8	-0.1, 1.7	0.8	-0.08, 1.7	0.4	-0.7, 1.5	**1.3**	**0.08, 2.4**	**1.3**	**0.2, 2.5**	0.8	-0.6, 2.1
	Winter	1.3	-0.1, 2.7	1.3	-0.05, 2.7	0.9	-0.7, 2.4	**2.0**	**0.07, 3.9**	**2.1**	**0.2, 3.9**	1.1	-1.0, 3.1
**Gardening**	Summer	**-0.4**	**-0.7, -0.2**	**-0.4**	**-0.6, -0.08**	**-0.5**	**-0.9, -0.2**	**-0.4**	**-0.8, -0.07**	**-0.4**	**-0.7, -0.01**	**-0.6**	**-1.0, -0.2**
	Winter	0.1	-0.4, 0.6	0.2	-0.3, 0.7	-0.2	-0.8, 0.4	-0.2	-0.9, 0.5	-0.1	-0.8, 0.5	-0.5	-1.2, 0.2

[a]Model 1 was adjusted for season and wear time at baseline and follow-up, and baseline total sedentary time.
[b]Model 2 was the same as model 1 plus mutually adjusted for age and sex.
[c]Model 3 was the same as Model 2 plus mutually adjusted for potential socioeconomic and environmental confounders (occupational class, educational level, job status, urban-rural status, smoking status, BMI).

Supplementary Table 2A. Complete case analysis.

Baseline Characteristic	Category/Unit	Total sedentary time (Min/day/yr)						Prolonged sedentary bouts (Min/day/yr)					
		Model 1		Model 2		Model 3		Model 1		Model 2		Model 3	
		β	95% CI	β	95% CI	β	95% CI	β	95% CI	β	95% CI	β	95% CI
**Sex**	Male (ref)												
	Female	**-3.1**	**-4.9, -1.4**	**-2.4**	**-4.1, -0.7**	**-2.5**	**-4.2, -0.7**	**-5.0**	**-7.3, -2.7**	**-4.2**	**-6.4, -1.9**	**-4.0**	**-6.3, -1.6**
**Age**	Per year of age	**0.6**	**0.5, 0.8**	**0.6**	**0.4, 0.7**	**0.6**	**0.4, 0.7**	**0.8**	**0.6, 1.0**	**0.8**	**0.6, 1.0**	**0.8**	**0.6, 1.1**
**Employment**	Yes (ref)												
	No	**2.8**	**0.7, 4.9**	0.7	-1.4, 2.9	0.7	-1.4, 2.9	**3.0**	**0.3, 5.8**	0.6	-2.3, 3.4	0.6	-2.2, 3.4
**Education level**	O level or less (ref)												
	A level or above	-0.8	-2.6, 1.0	-0.8	-2.5, 1.0	-0.6	-2.4, 1.2	0.9	-1.5, 3.3	0.7	-1.6, 3.0	0.7	-1.7, 3.1
**Smoking status**	Current (ref)												
	Former	0.4	-5.2, 6.0	-2.4	-7.9, 3.0	-3.4	-8.8, 2.1	2.1	-5.3, 9.4	-1.4	-8.5, 5.7	-2.9	-10.0, 4.2
	Never	-0.8	-6.3, 4.8	-3.2	-8.7, 2.2	-3.8	-9.3, 1.6	-0.1	-7.4, 7.2	-3.0	-10.0, 4.1	-4.2	-11.3, 2.8
**Body Mass Index**	Per kg/m^2	**0.3**	**0.07, 0.5**	**0.3**	**0.1, 0.5**	**0.4**	**0.1, 0.6**	**0.4**	**0.2, 0.7**	**0.5**	**0.2, 0.8**	**0.5**	**0.3, 0.8**
**Occupational classification**	Professional (ref)												
	Manager	0.1	-3.0, 3.1	0.1	-2.79, 3.07	-0.2	-2.94, 2.91	-2.2	-6.2, 1.8	-2.2	-6.1, 1.6	-2.3	-6.1, 1.5
	Skilled non-manual	-1.1	-4.7, 2.5	-0.3	-3.78, 3.11	-0.7	-4.18, 2.76	-1.7	-6.4, 3.0	-0.5	-5.0, 4.0	-0.5	-5.0, 4.0
	Skilled manual	0.5	-2.8, 3.8	0.8	-2.38, 4.06	0.6	-2.66, 3.84	-2.2	-6.6, 2.2	-1.9	-6.1, 2.3	-2.0	-6.2, 2.3
	Semi-skilled	0.5	-3.4, 4.5	0.5	-3.34, 4.29	0.0	-3.88, 3.85	-2.1	-7.3, 3.1	-2.1	-7.0, 3.0	-2.1	-7.2, 2.9
	Non-skilled	**8.5**	**1.9, 15.1**	**8.6**	**2.26, 15.0**	**8.0**	**1.59, 14.4**	4.3	-4.4, 13.1	3.8	-4.6, 12.2	3.5	-4.9, 11.9
**Urban-rural status**	City, town or fringe (Ref)												
	Village, hamlet or isolated dwelling	-1.4	-3.3, 0.5	-1.6	-3.46, 0.21	-1.8	-3.73, 0.06	-1.5	-3.9, 1.0	-1.6	-4.0, 0.8	-1.5	-3.9, 1.0

[a]Model 1 was adjusted for season and wear time at baseline and follow-up, and baseline total sedentary time.
[b]Model 2 was the same as model 1 plus mutually adjusted for age and sex.
[c]Model 3 was the same as Model 2 plus mutually adjusted for potential socioeconomic and environmental confounders (occupational class, educational level, job status, urban-rural status, smoking status, BMI).
Association of baseline demographic correlates with changes in total sedentary time and prolonged sedentary bouts (n=953).

Supplementary Table 2B. Complete case analysis.

Correlates	Category/Unit	Total sedentary time (Min/day/yr)						Prolonged sedentary bouts (Min/day/yr)					
		Model 1		Model 2		Model 3		Model 1		Model 2		Model 3	
		β	95% CI	β	95% CI	β	95% CI	β	95% CI	β	95% CI	β	95% CI
**Walking**	Per hour/week	-0.1	-0.2, 0.05	-0.1	-0.2, 0.02	-0.1	-0.2, 0.03	-0.1	-0.2, 0.07	-0.1	-0.2, 0.04	-0.1	-0.2, 0.06
**Cycling**	Per hour/week	-0.3	-0.6, 0.07	-0.3	-0.6, 0.02	-0.3	-0.6, 0.03	-0.3	-0.7, 0.3	-0.3	-0.8, 0.007	-0.3	-0.7, 0.1
**Garden**	Per hour/week	-0.1	-0.2, 0.09	**-0.2**	**-0.4, -0.09**	**-0.2**	**-0.4, -0.9**	-0.1	-0.3, 0.09	**-0.3**	**-0.4, -0.1**	**-0.3**	**-0.4, -0.1**
**Housework**	Per hour/week	0.0	-0.06, 0.09	0.1	-0.02, 0.1	0.1	-0.03, 0.1	0.0	-0.1, 0.06	0.0	-0.07, 0.1	0.0	-0.07, 0.1
**Dog walking**	No (ref)												
	Yes	-1.2	-3.3, 1.0	-0.8	-2.9, 1.3	-0.9	-3.0, 1.3	-0.6	-3.4, 2.2	0.3	-2.4, 3.0	0.4	-2.3, 3.2
**Transport method <1 mile**	Car (ref)												
	Walk	2.0	-0.4, 4.4	1.0	-1.4, 3.3	1.2	-1.1, 3.6	**3.6**	**0.5, 6.8**	2.3	-0.7, 5.4	2.9	-0.2, 5.9
	Public transport	6.2	-4.7, 17.0	3.1	-7.4, 13.7	3.5	-7.0, 13.9	4.3	-10.0, 18.6	1.2	-12.5, 14.9	2.3	-11.4, 15.9
	Cycle	1.2	-3.0, 5.4	-0.2	-4.3, 3.9	0.2	-3.9, 4.3	1.5	-4.0,7.0	-0.2	-5.5, 5.1	0.7	-4.7, 6.0
**Transport method 1-5 miles**	Car (ref)												
	Walk	-0.2	-3.1, 2.7	-0.5	-3.3, 2.3	-0.3	-3.1, 2.6	-0.1	-3.9, 3.7	-0.3	-4.0, 3.3	0.1	-3.6, 3.8
	Public transport	0.6	-2.1, 3.2	-0.2	-2.8, 2.4	-0.3	-2.9, 2.4	1.1	-2.4, 4.6	0.1	-3.3, 3.5	0.2	-3.3, 3.6
	Cycle	1.0	-2.0, 3.9	0.4	-2.4, 3.3	0.6	-2.2, 3.5	-1.5	-5.3, 2.4	-2.0	-5.7, 1.7	-1.4	-5.1, 2.3
**Transport method >5 miles**	Car (ref)												
	Walk	7.9	-10.6, 26.4	4.3	-13.6, 22.2	4.5	-13.2, 22.3	5.6	-18.8, 29.9	1.6	-21.7, 25.0	2.7	-20.6, 25.9
	Public transport	0.3	-3.4, 3.9	-0.7	-4.2, 2.8	-0.6	-4.2, 2.9	-2.1	-6.9, 2.6	-3.5	-8.1, 1.1	-3.3	-7.9, 1.3
	Cycle	-10.9	-20.8, 1.0	-11.9	-21.6, 2.3	-13.6	-23.2, 4.0	-4.1	-17.1, 9.0	-5.4	-17.9, 7.2	-7.5	-20.0, 5.1
**TV**	Per hour/week	0.6	-0.01, 1.1	0.4	-0.1, 1.0	0.2	-0.4, 0.8	0.4	-0.3, 1.2	0.3	-0.4, 1.0	0.2	-0.6, 1.0
**Radio**	≤Several times/yr (ref)												
	Several times/month	-1.9	-5.0, 1.2	-2.2	-5.2, 0.7	-2.2	-5.2, 0.7	-0.8	-4.9, 3.3	-1.1	-5.0, 2.8	-0.9	-4.8, 3.0
	≥Several times/week	-1.8	-4.1, 0.5	-2.2	-4.5, 0.02	-1.9	-4.1, 0.4	-0.5	-3.5, 2.6	-1.0	-3.9, 1.9	-0.6	-3.5, 2.3
**Newspaper**	≤Several times/yr (ref)												
	Several times/month	0.1	-4.1, 4.4	0.5	-3.6, 4.7	1.2	-2.9, 5.3	-2.8	-8.4, 2.8	-1.9	-7.3, 3.5	-1.5	-6.9, 3.9
	≥Several times/week	2.2	-1.3, 5.6	1.1	-2.2, 4.5	1.7	-1.7, 5.0	1.4	-3.1, 5.9	0.1	-4.3, 4.5	0.4	-3.9, 4.8
**Books**	≤Several times/yr (ref)												
	Several times/month	-0.8	-3.8, 2.3	-0.3	-3.2, 2.7	0.2	-2.8, 3.2	-1.3	-5.4, 2.7	-0.4	-4.2, 3.6	0.1	-3.8, 4.0
	≥Several times/week	-1.8	-3.8, 0.2	-1.5	-3.4, 0.5	-1.1	-3.1, 0.9	-2.5	-5.1, 0.06	-1.8	-4.3, 0.7	-1.6	-4.1, 1.0
**Computer use**	Per hour/week	-0.7	-1.45, 0.04	-0.3	-1.1, 0.4	-0.1	-0.9, 0.7	-0.1	-0.9, 1.0	0.5	-0.5, 1.5	0.6	-0.5, 1.6

Association of baseline behavioural correlates with changes in total sedentary time and prolonged sedentary bouts (n=953).

Supplementary Table 2C. Complete case analysis.

Change in Correlate	Category/ Unit	Total sedentary time (Min/day/yr)						Prolonged sedentary bouts (Min/day/yr)					
		Model 1		Model 2		Model 3		Model 1		Model 2		Model 3	
		β	95% CI	β	95% CI	β	95% CI	β	95% CI	β	95% CI	β	95% CI
**Employment change**	Remains retired (Ref)												
	Becomes employed	1.2	-7.1, 9.4	0.6	-7.5, 8.6	0.9	-7.1, 9.0	3.5	-7.6, 14.6	2.4	-8.4, 13.1	2.7	-8.1, 13.5
	Remains employed	**4.0**	**1.0, 7.0**	2.0	-1.0, 5.0	1.8	-1.3, 4.8	4.0	-0.1, 8.1	1.3	-2.8, 5.4	0.9	-3.2, 5.1
	Becomes retired	1.5	-2.4, 5.3	2.1	-1.6, 5.9	1.3	-2.5, 5.0	2.1	-3.1, 7.2	2.9	-2.1, 7.9	2.1	-3.0, 7.2
**Body Mass Index**	Per kg/m^2/yr	**4.8**	**2.3, 7.4**	**5.2**	**2.6, 7.7**	**5.7**	**3.2, 8.2**	2.6	-0.9, 6.1	3.2	-0.2, 6.6	**3.1**	**3.9, 1.7**
**Walking**	**Per hour/week/yr**	**-0.7**	**-1.0, -0.3**	**-0.7**	**-1.0, -0.3**	**-0.8**	**-1.1, -0.4**	-0.3	-0.8, 0.2	-0.3	-0.8, 0.1	-0.4	-0.9, 0.2
**Cycling**	**Per hour/week/yr**	1.1	-0.3, 2.4	0.9	-0.4, 2.3	0.2	-1.3, 1.8	**2.4**	**0.6, 4.2**	**2.2**	**0.5, 4.0**	0.9	-1.0, 2.9
**Gardening**	**Per hour/week/yr**	**-0.9**	**-1.5, -0.3**	**-0.7**	**-1.3, -0.2**	**-1.2**	**-1.8, -0.5**	-0.8	-1.6, 0.02	-0.6	-1.4, 0.2	**-1.0**	**-0.9, -0.1**
**Housework**	**Per hour/week/yr**	-0.3	-0.6, 0.02	-0.3	-0.7, 0.2	-0.3	-0.6, 0.08	-0.2	-0.6, 0.2	-0.3	-0.7, 0.2	-0.2	-0.6, 0.3
**TV**	**Per hour/week/yr**	**4.0**	**0.4, 7.7**	**4.3**	**0.7, 7.8**	**4.5**	**0.7, 8.3**	3.4	-1.6, 8.3	3.7	-1.1, 8.5	4.5	-0.8, 9.8

[a]Model 1 was adjusted for season and wear time at baseline and follow-up, and baseline total sedentary time.
[b]Model 2 was the same as model 1 plus mutually adjusted for age and sex.
[c]Model 3 was the same as Model 2 plus mutually adjusted for potential socioeconomic and environmental confounders (occupational class, educational level, job status, urban-rural status, smoking status, BMI).
Association of change in correlates with change in total sedentary time and prolonged sedentary bouts (n=856).
